# Luteinizing hormone profiles during ovarian stimulation in assisted reproductive treatment

**DOI:** 10.3389/fendo.2024.1481546

**Published:** 2024-12-04

**Authors:** Hannah Verschuere, Annouschka Laenen, Sophie Debrock, Carla Tomassetti, Sharon Lie Fong

**Affiliations:** ^1^ Leuven University Fertility Center, Department of Gynecology and Obstetrics, University Hospitals Leuven, Leuven, Belgium; ^2^ Fertility Clinic Brussels, Clinic St. Jean Brussels, Brussels, Belgium; ^3^ Leuven Center for Biostatistics and Statistical Bioinformatics, KU Leuven, Leuven, Belgium; ^4^ Laboratory of Endometrium, Endometriosis and Reproductive Medicine, Department of Development and Regeneration, KU Leuven, Leuven, Belgium

**Keywords:** luteinizing hormone (LH), ovarian stimulation, antagonist, oversuppression, hormonal profile

## Abstract

**Introduction:**

Few data is available on the natural course of luteinizing hormone (LH) during ovarian stimulation, but it has been suggested that ‘oversuppressed’ LH could decrease fertility outcomes. Our aim with this study is to evaluate the changes in LH depending on the used stimulation protocol to better define LH oversuppressioin.

**Methods:**

Patients undergoing oocyte retrieval in a tertiary fertility center between 01-01-2015 and 30-09-2020 after stimulation with a short-agonist (SA) or antagonist (A) protocol were included. Data were retrospectively retrieved from 858 electronic patient records, of which 338 SA cycles and 783 A cycles. A continuous profile was set out to evaluate the pooled measurements of the mean LH in time during ovarian stimulation and linear mixed modeling was used to compare the change of LH between 4 time points: the day prior to start of gonadotrophins (T1), stimulation day 5 (T2), stimulation day 6 (T3) and on the day of oocyte maturation trigger (T4). Oversuppression of LH was defined as a decrease of LH > 50% after initiation of GnRH antagonist and LH levels < 1.2 IU/l after GnRH antagonist. A subanalysis was performed for type of gonadotrophin (recFSH vs hp-hMG).

**Results:**

In the SA protocol, an initial LH peak was followed by a slow decrease of LH until triggering. In the A protocol, LH decreased after gonadotrophin initiation with a further rapid decrease after initiation of the antagonist and remained low until trigger. LH levels dropped > 50% in 26.2% of the antagonist cycles and LH levels were < 1.2 IU/l in 45.3% of cycles after initiation of GnRH-antagonist.

**Conclusion:**

The course of LH in the SA protocol differs from the A protocol where low mean LH levels are seen. Oversuppression of LH, or iatrogenic LH deficiency as described in earlier studies, may be a rather pervasive phenomenon during stimulation with an antagonist protocol and warrants further investigation to elucidate the clinical relevance of low LH levels during ovarian stimulation.

## 1 Introduction

Luteinizing hormone (LH) is a glycoprotein, secreted by the anterior pituitary gland in a pulsatile fashion with variation in frequency and amplitude according to the phase of the menstrual cycle (1). It has a wide range of functions during different stages of the cycle and holds an important role in follicle maturation and maintaining corpus luteum function during the luteal phase. It also plays a key role in the early and mid-follicular phase in promoting steroidogenesis and follicle development (2). In an ovulatory cycle, LH levels are low during the early follicular phase but start to rise in the mid-follicular phase as a response to the rising estrogen levels, and eventually increase to the LH peak prior to ovulation. During controlled ovarian stimulation (COS) in assisted reproductive treatments (ART), i.e. *in vitro* fertilization (IVF) and intra cytoplasmatic sperm injection (ICSI), exogenous FSH administration ensures follicle growth, either with recombinant FSH (rec-FSH) or with highly purified human menopausal gonadotrophin (hp-hMG) (3, 4). In contrast, standard LH supplementation does not seem to be a prerequisite for successful oocyte retrieval (5). Gonadotrophin releasing hormone (GnRH) agonists have been introduced to inhibit a premature LH surge to optimize IVF treatment outcome. GnRH-agonists cause an initial flare-up of follicle stimulating hormone (FSH) and LH secretion and thereafter suppress gonadotrophin secretion by downregulation (6). Several studies have described lower life birth rates after profound suppression of LH levels during COS with long agonist protocol (7–9). These studies suggest that this iatrogenic LH deficiency may lead to an intrafollicular environment with reduced concentrations of serum estradiol which might lead to suboptimal maturation for a normal oocyte during the follicular phase.

Nowadays, the use of COS with an GnRH antagonist is recommended with regard to prevention of ovarian hyperstimulation syndrome (10). The GnRH antagonist will block the GnRH receptor without a flare-up effect and will cause a decrease of LH levels, whereas FSH levels will be less affected (11). Few studies have evaluated the effect of LH levels during controlled ovarian stimulation on clinical outcome in antagonist cycles and these show conflicting results (12–15). Consequently, it is unclear whether profoundly suppressed endogenous LH levels may impact treatment outcome and how to define this so-called ‘over-suppression’ of LH. Moreover, there is limited information on the course of LH levels during COS in antagonist stimulation protocols. Nevertheless, several studies have suggested that some women may benefit from LH supplementation during COS (2, 16, 17). Until so far, conclusive data on possible candidates for such treatment are lacking (18). Therefore, the aim of our study was to describe the profile of LH during COS with short GnRH agonist and with GnRH antagonist. We also compared LH levels during COS with rec-FSH and with hp-hMG.

## 2 Methods

### 2.1 Study population

This study was a retrospective study in patients who underwent ART treatment at a tertiary level infertility clinic, at the Leuven University Fertility Center between 01/01/2015 and 31/09/2020. Clinical and treatment-related data was extracted from the patients’ electronic medical records. All ovarian stimulation cycles with a short agonist (SA) protocol and antagonist protocol (A) were screened. Only cycles with serial hormonal blood sampling including baseline blood sampling were eligible. Since we aimed to describe LH profiles during a standard controlled ovarian stimulation with a short-agonist protocol or antagonist protocol, the following cycles were excluded: stimulation with a long-agonist protocol; cycle cancellation prior to oocyte retrieval; preparation with oral contraceptive pills; concomitant use of clomiphene citrate, aromatase inhibitor or hormonal IUD; dual stimulations; stimulation duration shorter than 9 days or longer than 16 days.

### 2.2 Primary outcome

Our aim was to describe the change of endogenous LH levels in patients undergoing ovarian stimulation for ART with respect to the effect of GnRH analogues during a standard stimulation. We set out the course of LH from start of ovarian stimulation until oocyte maturation triggering.

### 2.3 Measurements

**Figure 1:**
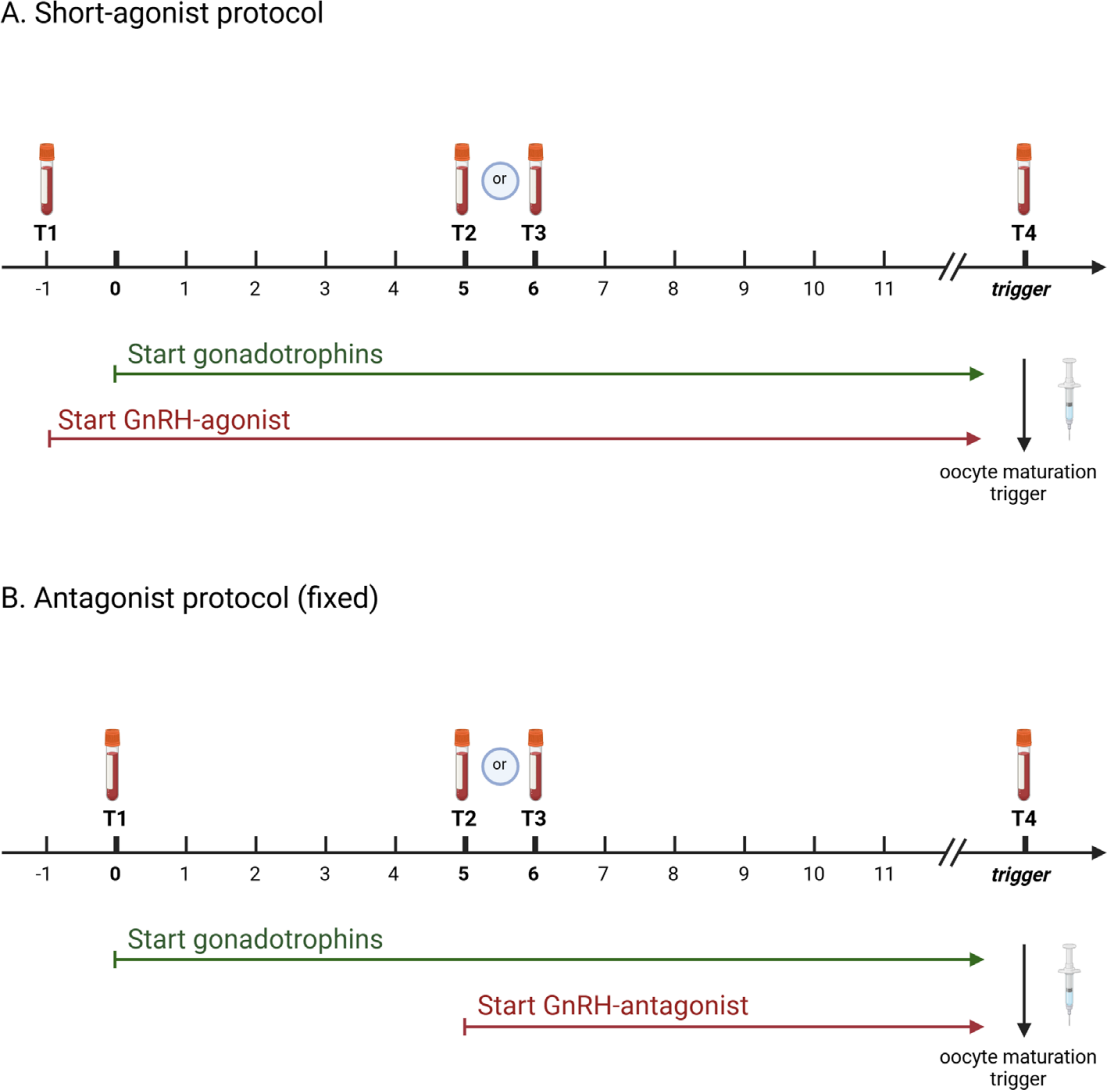
Time line of study protocol indicating time points of blood sampling during ovarian stimulation in function of gonadotrophin administration in short-agonist protocol **(A)** and antagonist protocol **(B)**. Created with BioRender.com.

A timeline was set out in function of gonadotrophin administration, defining day 0 as the first administration of FSH. A baseline blood testing was taken between menstrual cycle day 1 to 3, i.e. one day prior to or on the day of start of gonadotrophin stimulation. Subsequently, blood samples were taken during ovarian stimulation and the day before or on the day of oocyte maturation triggering for oocyte retrieval. Consequently, four time points were identified at which LH levels were measured (**Figure 1**). The time line in the two studied stimulation protocols was as follows:

• Short agonist (SA): treatment is initiated at the beginning of the menstrual cycle day 1 to 3 with a GnRH agonist and gonadotrophin administration is started the day after (**Figure 1A**)
 o T1: ‘baseline’: between menstrual cycle day 1 to 3 a blood sample was taken prior to the first injections. This was the day of GnRH agonist initiation (day -1) and 1 day before the first gonadotrophin administration
 o T2: this blood sample was taken on day 5 of the gonadotrophin administration (6th administration of gonadotrophins)
 o T3: this blood sample was taken on day 6 of gonadotrophin administration (7th administration of gonadotrophins)
 o T4: this blood sample was taken on the day of oocyte maturation trigger
• Antagonist (A): treatment is start at the beginning of the menstrual cycle day 1 to 3 with initation of Gonadotrophins. The GnRH antagonist is initiated on the 6th day of gonadotrophin administration, i.e. a fixed protocol (**Figure 1B**)
 o T1: ‘baseline’: between menstrual cycle day 1 to 3 a blood sample was taken prior to the first injections. Gonadotrophins were initiated on that same day (day 0).
 o T2: this blood sample was taken on day 5 of gonadotrophin administration (6th administration of gonadotrophins). The GnRH antagonist is initiated on that day.
 o T3: this blood sample was taken on day 6 of gonadotrophin administration (7th administration of gonadotrophins) and approximately 24 hours after the first GnRH antagonist injection.
 o T4: this blood sample was taken on the day of oocyte maturation trigger

LH levels were measured immediately after blood sample collection. LH and AMH measurements were performed with ECLIA by Roche diagnostics.

### 2.4 Statistical analysis

Patient characteristics were described, but due to the exploratory nature of this study, characteristics of patients included in the short agonist (SA) protocol were not compared with those of patients in the antagonist (A) group. Mean LH with 95% confidence interval was set out as a continuous, linear profile to evaluate changes over time during stimulation. In addition, descriptive analyses of LH levels were performed at each time point T1, T2, T3 and T4. Finally, linear mixed models (LMM) were used to compare LH Levels between the time points. Random effect for patient and cycle were modelled to deal with clustering. We evaluated the estimated mean LH levels based on a longitudinal model. To account for the skewed distribution of the variables, a log transformation was performed to adjust for the influence of outliers. Change in LH between time points was expressed as the ratio of the later value over the earlier value. A ratio < 1.00 indicated decrease, whereas a ratio > 1.00 indicated increase of LH.

A sub-analysis was performed to evaluate the possible effect of the type of gonadotrophin used on the mean LH levels during COS and after linear mixed models. To identify patients with so-called ‘oversuppressed’ LH levels in the antagonist group, a selection was made of cycles with blood samples performed at both T2 and T3 to evaluate the decrease of LH after GnRH antagonist initiation. Two cut-off levels used in earlier studies to define ‘oversuppression’ were used in this study: a decrease of > 50% between blood samples taken prior to GnRH antagonist administration (T2) and 24 hours after GnRH antagonist administration (T3) (9, 17) and LH levels during ovarian stimulation < 1.2 IU/L at T2 and T3 (9, 19). An additional subanalysis was performed to evaluate possible confounding factors on LH levels (AMH, BMI and age) using a logistic regression analysis to calculate odds ratio with 95% confidence intervals. We applied the same aforementioned cut-off levels as mentioned above to identify cycles with so-called ‘oversuppressed’ LH levels, rather than using a cut-off levels based on e.g. percentiles or quartiles in the current cohort. The odds ratio represented the impact of a 1-unit increase of the predictor on ‘oversuppression’. An odds ratio < 1 indicated a lower probability of LH < 1.2 IU/l at T3 or >50% decrease of LH between T2 and T3. An odds ratio > 1 indicated a higher probability of LH < 1.2 IU/l at T3 or >50% decrease of LH between T2 and T3. We reported pregnancy rates, i.e. positive pregnancy test, after fresh embryo transfer in both groups according to both definitions of oversuppression. Since this was a descriptive study, no power calculation was performed to detect any difference in the number of ‘oversuppressed’ levels or pregnancy rates between the studies groups, neither for the differences in type of gonadotrophin.

Statistical analysis was performed using SAS software version 9.4. Level of significance was set at P < 0.05.

## 3 Results

A total of 1180 cycles were analyzed in 858 patients. Most cycles (842; 71.4%) were performed with an antagonist (A) protocol, 338 cycles (28.6%) were performed with a short-agonist (SA) protocol. In 783 cycles rec-FSH was used (66.4%), compared to hp-hMG in 397 cycles (33.6%).

The mean age of women in the SA group was 37.0 years (± 4.5 years), whereas the mean age in the A group was 33.1 years (± 5.0 years). Similarly, mean serum AMH in the SA group was 1.2 ng/ml (± 1.2 ng/ml) and 3.2 ng/ml (± 3.3 ng/ml) in the A group. Body mass index was 25.2 kg/m² (± 4.6 kg/m²) in the SA group and 24.3 kg/m² (± 4.3 kg/m²) in the A group. The mean duration of COS was 11.5 days (± 1.9 days) in the SA group, and 11.9 days (± 1.9 days) in the A group. The mean starting dose of gonadotrophins was 231 IU (± 43 IU) with a mean total gonadotrophins dose of 2262 IU (± 669 IU) during stimulation in a short-agonist protocol. In the antagonist group, mean starting dose was lower (187 ± 54 IU) and total mean gonadotrophins dose was 1880 IU (± 627 IU). In the short-agonist group the mean number of oocytes able to retrieve was 5.9 (± 4.3 oocytes), 237 fresh embryo transfers were performed of which 48 had positive HCG test (20.3%). In the antagonist group the mean number of oocytes able to retrieve was 10.4 (± 7.6 oocytes). In this group, 443 embryo transfers were performed of which 146 had a positive HCG test (33.0%) (**Table 1**).

**Table 1:** Patient characteristics and characteristics of stimulation protocol.

A: Patient characteristics				
	Short-agonist		Antagonist	
Mean age (years; SD)	37.0 (± 4.5)		33.1 (± 5.0)	
Anti-Müllerian hormone (ng/ml; SD)	1.2 (± 1.2)		3.2 (± 3.3)	
Body mass index (kg/m^2^; SD)	25.2 (± 4.6)		24.3 (± 4.3)	
B: Stimulation characteristics				
	Short-agonist		Antagonist	
Number of cycles (n:N %)	338/1180	(28.6%)	842/1180	(71.4%)
- Recombinant FSH	181/1180	(15.3%)	602/1180	(51.0%)
- Hp-hMG	157/1180	(13.3%)	240/1180	(20,.3%)
Duration stimulation (days; SD)	11,5	(± 1.9)	11,9	(± 1.9)
Starting dose FSH (units; SD)	231	(± 43)	187	(± 54)
Total dose FSH (units; SD)	2262	(± 669)	1880	(± 627)
Number of oocytes retrieved (n; SD)	5,9	(± 4.3)	10,4	(± 7.1)
Number of fresh embryotransfer (n/N; %)	237/338	(70.1%)	443/842	(53.6%)
Outcome embryotransfer (n/N; %)				
- HCG detected	48/237	(20.3%)	146/443	(33.0%)
- No HCG detected	187/237	(78.9%)	297/443	(67.0%)
- Loss of follow-up	2/237	(0.8%)	0/443	(0 %)

### 3.1 Type of COS protocol

**Figure 2:**
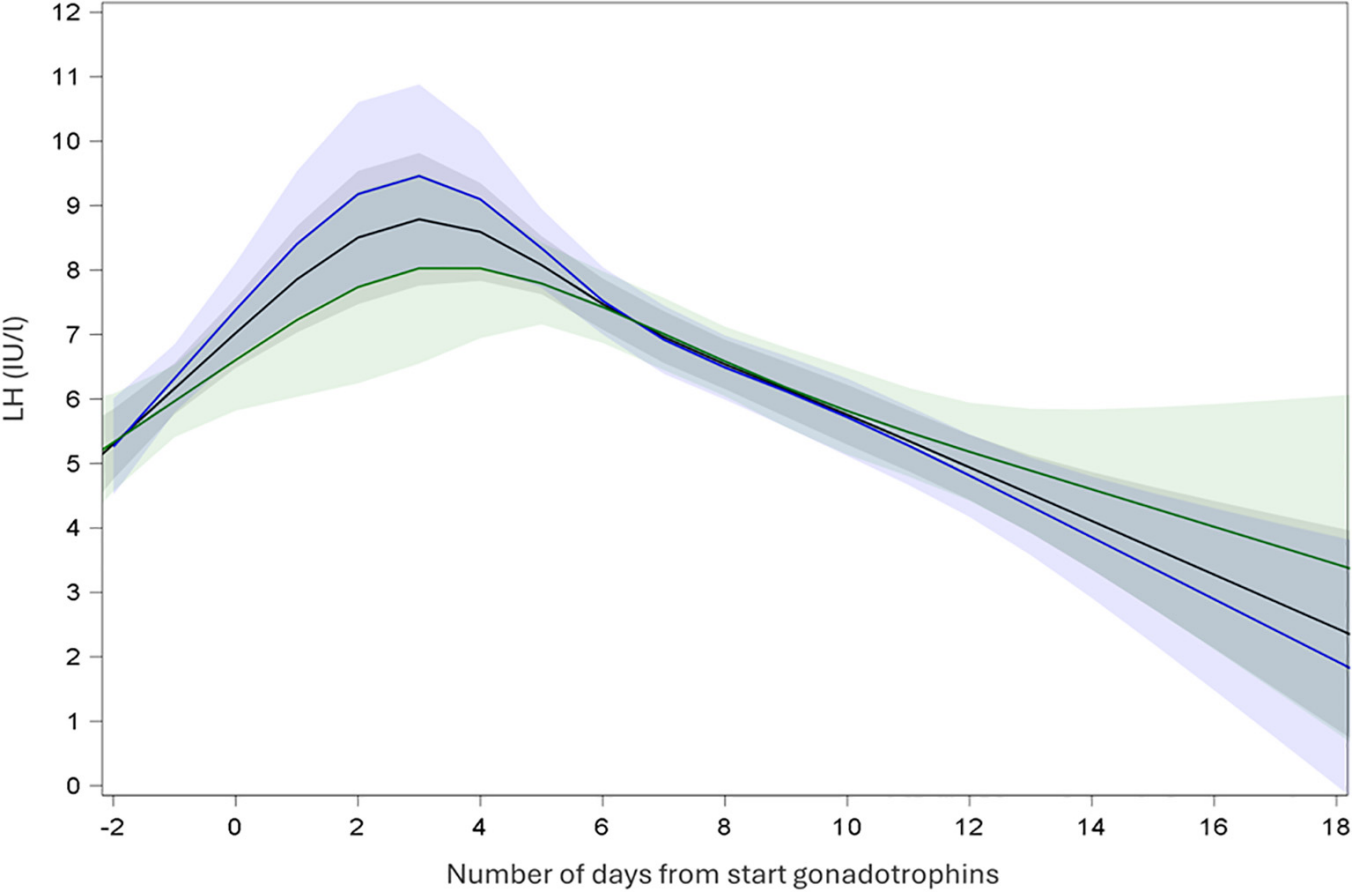
Endocrine profile of mean LH with 95% confidence interval during ovarian stimulation in short-agonist protocol. Black: all cycles; Blue: recombinant gonadotrophins only; Green: highly purified human menopausal gonadotrophin only.

**Figure 3:**
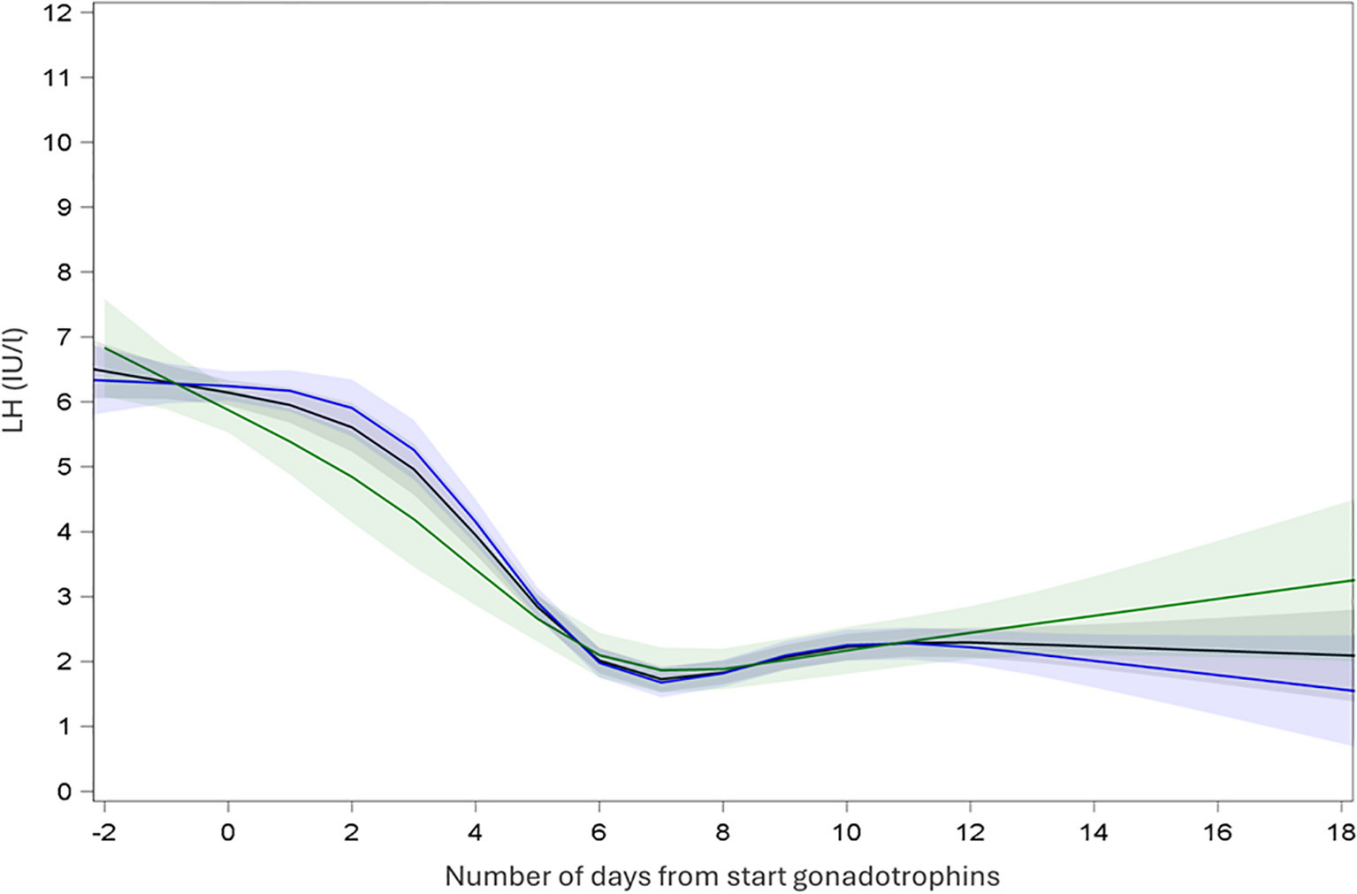
Endocrine profile of mean LH with 95% confidence interval during ovarian stimulation in antagonist protocol. Black: all cycles; Blue: recombinant gonadotrophins only; Green: highly purified human menopausal gonadotrophin only.

In SA cycles, continuous measurements of LH showed an initial increase of LH levels after initiation of GnRH agonist and a subsequent decrease of LH until oocyte maturation trigger prior to oocyte retrieval (**Figure 2**). Continuous measurements of LH over time in A cycles showed a steady decrease of LH after start of gonadotrophins and a steep decrease of LH after GnRH antagonist initiation. LH remained low until oocyte maturation trigger for the oocyte retrieval (**Figure 3**).

In SA cycles, mean LH was measured at specific time points during ovarian stimulation (**Table 2**). After performing LMM analyses, mean LH at baseline (T1) was LH 4.45 IU/l (CI: 4.10; 4.82), at T2 mean LH 6.71 IU/l (CI: 6.04; 7.47), at T3 6.36 IU/l (CI: 5.85;6.91) and at T4 5.59 (5.16; 6.06) (**Table 3**). Using LMM analysis, changes in LH levels as compared to baseline LH (T1) were calculated; ratio T2:T1 was 1.51 (CI 1.66; 1.37; P < 0,001) and ratio T3:T1 was 1.43 (CI: 1.54;1.33; P < 0,001). As compared to the 5th stimulation day (T2), LH levels did not change significantly [ratio T3:T2 0.95 (1.05; 0, 86; P = 0,29)], whereas ratio T4:T2 (ratio 0.83; CI 0.92; 0.76; P < 0.001) and ratio T4:T3 (ratio 0.88; CI 0.95; 0.82; P < 0.001) confirmed a significant change in estimated LH levels until oocyte maturation trigger (**Figure 2**, **Table 4**).

**Table 2:** Measured mean LH levels (IU/l) for the short-agonist and antagonist protocol at the different set time points.

Time point (TP)	Agonist-short		Antagonist	
	Mean LH (IU/l)	SD	Mean LH (IU/l)	SD
T1	5,97	5,71	6,32	3,16
T2	7,43	3,48	2,80	3,52
T3	7,20	3,05	1,83	2,36
T4	6,15	2,70	2,24	2,69

**Table 3:** Mean LH levels (IU/l) for the short-agonist and antagonist protocol at the different set time points after LMM analysis.

A: all gonadotrophins				
Time point (TP)	Agonist-short		Antagonist	
	Mean LH (IU/l)	95% CI	Mean LH (IU/l)	95% CI
T1	4.45	(4.10;4.81)	5.36	(5.03;5.72)
T2	6.71	(6.04;7.47)	1.85	(1.73;1.99)
T3	6.36	(5.85;6.91)	1.22	(1.14;1.31)
T4	5.59	(5.16;6.06)	1.30	(1.22;1.39)
B: rec-FSH				
Time point (TP)	Agonist-short		Antagonist	
	Mean LH (IU/l)	95% CI	Mean LH (IU/l)	95% CI
T1	4.55	(4.10;5.04)	5.43	(5.03;5.87)
T2	6.91	(5.99;7.98)	1.81	(1.66;1.97)
T3	6.53	(5.87;7.28)	1.21	(1.11;1.32)
T4	5.45	(4.92;6.05)	1.29	(1.20;1.40)
C: hp-hMG				
Time point (TP)	Agonist-short		Antagonist	
	Mean LH (IU/l)	95% CI	Mean LH (IU/l)	95% CI
T1	4.29	(3.81;4.83)	5.23	(4.68;5.85)
T2	6.42	(5.51;7.48)	1.97	(1.74;2.22)
T3	6.09	(5.38;6.90)	1.26	(1.12;1.43)
T4	5.69	(5.05;6.41)	1.32	(1.18;1.48)

A. All gonadotrophins. B. Rec-FSH. C. hp-hMG.

**Table 4:** Ratio of change in LH levels between time points.

A: Short-agonist									
*TP*	All gonadotrophins			Rec-FSH			Hp-hMG		
	Ratio (CI 95%)	*p-value*		Ratio (CI 95% )	*p-value*		Ratio (CI 95% )	*p-value*	
T1 vs T2	1.51 (1.66;1.37)	< 0.01	↑	1.52 (1.74;1.33)	< 0.01	↑	1.50 (1.72;1.31)	< 0.01	↑
T1 vs T3	1.43 (1.54;1.33)	< 0.01	↑	1.44 (1.59;1.30)	< 0.01	↑	1.42 (1.58;1.28)	< 0.01	↑
T1 vs T4	1.26 (1.35;1.17)	< 0,01	↑	1.20 (1.32;1.09)	< 0.01	↑	1.33 (1.47;1.20)	< 0.01	↑
T2 vs T3	0.95 (1.05;0.85)	0.30	=	0.95 (1.09;0.82)	0.43	=	0.95 (1.10;0.81)	0.48	=
T2 vs T4	0.83 (0.92;0.76)	< 0.01	↓	0.80 (0.90;0.69)	< 0.01	↓	0.89 (1.02;0.77)	0.09	=
T3 vs T4	0.88 (0.95;0.82)	< 0.01	↓	0.84 (0.92;0.76)	< 0.01	↓	0.93 (1.04;0.84)	0.20	=
B: Antagonist									
*TP*	All gonadotrophins			Rec-FSH			Hp-hMG		
	Ratio (CI 95%)	*p-value*		Ratio (CI 95% )	*p-value*		Ratio (CI 95% )	*p-value*	
T1 vs T2	0.35 (0.37;0.33)	< 0.01	↓	0.33 (0.36;0.31)	< 0.01	↓	0.38 (0.42;0.34)	< 0.01	↓
T1 vs T3	0.23 (0.24;0.21)	< 0.01	↓	0.22 (0.24;0.21)	< 0.01	↓	0.24 (0.27;0.22)	< 0.01	↓
T1 vs T4	0.24 (0.26;0.23)	< 0.01	↓	0.24 (0.26;0.22)	< 0.01	↓	0.25 (0.27;0.23)	< 0.01	↓
T2 vs T3	0.67 (0.71;0.62)	< 0.01	↓	0.67 (0.73;0.61)	< 0.01	↓	0.64 (0.72;0.57)	< 0.01	↓
T2 vs T4	0.70 (0.75;0.66)	< 0.01	↓	0.72 (0.77;0.66)	< 0.01	↓	0.67 (0.75;0.60)	< 0.01	↓
T3 vs T4	1.07 (0.35;1.00)	0.05	↑	1.07 (1.16;0.99)	0.08	=	1.05 (1.16;0.94)	0.41	=

A. Short-agonist protocol. B. Antagonist protocol. Arrow up: indicates significant increase of LH; Arrow down: indicates significant decrease of LH; = : indicates no significant change.

Similarly, mean LH was measured in A cycles (**Table 2**). Subsequently LMM analysis was performed which showed a mean LH at T1 5.36 IUI/l (CI: 5.03; 5.72), T2 of 1.85 IU/l (CI: 1.73; 1.99), T3 of 1.22 IU/l (CI: 1.14; 1.31) and T4 of 1.30 IU/l (CI: 1.22; 1.39) (**Table 3**). Significant changes in LH level were observed between T1 and T2 [ratio T2:T1 0.35 (CI: 0.37; 0.33; P < 0.001)], between T1 and T3 (ratio 0.23; CI: 0.24; 0.21; P < 0.001) and between T1 and T4 (ratio 0.24; CI: 0.26; 0.23; P < 0.001). Mean LH levels also changed significantly between T2 and T3 (ratio 0.66 (CI: 0.71; 0.62; P < 0,001) and between T2 and T4 (ratio 0.70; CI: 0.75;0.66; P < 0.001). Finally, a significant change in mean LH was also observed between T3 and T4 (ratio 1.06; CI: 1.14; 1.00; P < 0,05) (**Figure 3**, **Table 4**).

### 3.2 Type of gonadotrophin

In each COS protocol, changes in mean LH levels were also calculated in cycles using rec-FSH and in cycles using hp-hMG. In SA cycles, for the group using rec-FSH, LH at baseline (T1) was LH 4.55 IU/l (CI: 4.10; 5.04), at T2 mean LH 6.91 IU/l (CI: 5.99; 7.98), at T3 6.53 IU/l (CI: 5.87;7.28) and at T4 5.45 (4.92; 6.05) (**Table 3**). In these cycles using rec-FSH, mean LH changed significantly between all time points, except between T2 and T3 [ratio 0.95 (CI: 1.09;0.82; P = 0.43)] (**Figure 2**, **Table 4**). For the group using hp-hMG LH at baseline (T1) was LH 4.29 IU/l (CI: 3.81; 4.83), at T2 mean LH 6.42 IU/l (CI: 5.51; 7.48), at T3 6.09 IU/l (CI: 5.38;6.90) and at T4 5.69 (5.05; 6.41) (**Table 3**). In cycles with hp-hMG in the SA protocol, mean LH was significantly different in all time points as compared to T1. However, none of the other comparisons reached statistical significance; T2 vs T3 (ratio 0.95 (CI: 1.1;0.82; P = 0.48), T2 vs T4 (ratio 0.89 (CI: 1.02; 0.77; P = 0.09) and T3 vs T4 (ratio 0.93 (CI: 1.04; 0.84; P = 0.20) (**Figure 2**, **Table 4**).

In cycles in the antagonist protocol with rec-FSH, mean LH at T1 was 5.43 IUI/l (CI: 5.03; 5.87), T2 of 1.81 IU/l (CI: 1.66; 1.97), T3 of 1.21 IU/l (CI: 1.11; 1.32) and T4 of 1.29 IU/l (CI: 1.20; 1.40) (**Table 3**). In this group, using rec-FSH, mean LH changed significantly between all time points, except between T3 and T4 (ratio 1.07 (CI: 1.16;0.99; P = 0.08) (**Figure 3**, **Table 4**). Similarly, in cycles with hp-hMG, T1 was 5.23 IUI/l (CI: 4.68; 5.85), T2 of 1.97 IU/l (CI: 1.74; 2.22), T3 of 1.26 IU/l (CI: 1.12; 1.43) and T4 of 1.32 IU/l (CI: 1.18; 1.48) (**Table 3**), and only the change in mean LH levels between T3 vs T4 was not significantly different (ratio 1,05 (CI: 1,16; 0,94; P = 0,40) (**Figure 3**, **Table 4**). Thus LMM showed a similar mean LH profile in rec-FSH as compared to hp-hMG cycles for antagonist cycles.

### 3.3 Oversuppression

In 401 antagonist cycles a blood sample was taken at both time points T2 and T3, i.e. after GnRH antagonist initiation. In 105 (26.2%) cycles a drop of > 50% of LH was observed, indicating possible oversuppression of LH. A drop of > 50% of LH was observed in 77 cycles (77/286; 26.9%) using rec-FSH in an antagonist protocol, and in 28 cycles (28/119; 24.4%) for hp-hMG (**Table 5**). Logistic regression analysis to evaluate possible confounding factors for LH oversuppression with a > 50% decrease of LH between T2 and T3 showed a significant odds ratio for age (OR 0.95 (0.90;0.99), P 0.02) and BMI (OR 0.94 (0.88;0.99), P = 0.03), whereas AMH had no impact (OR 1.03 (0.96;1.10), P = 0.48) (**Table 6**). In 216/401 cycles a fresh embryo transfer was performed resulting in a pregnancy rate of 25.6% (11/43) in the oversuppressed group compared to 38.7% (67/173) in the group where the decrease in LH was < 50% between T2 and T3. After using rec-FSH, pregnancy test was positive in 22.6% (7/31) of cycles in the oversuppressed group, compared to 44.4% (51/115) in the group where LH dropped < 50%. Using hp-hMG, pregnancy rates in the oversuppressed group were 33.3% (4/12) and 27.6% (15/58) in the group with LH decrease < 50% (**Table 7**).

**Table 5:** Oversuppression in antagonist cycles.

A: all gonadotrophins			
	Statistics	T2 vs. T3 > 50% decrease LH	LH < 1.2 IU/l at T3
No	n/N (%)	296/401 (73.8%)	329/601 (54.7%)
Yes	n/N (%)	105/401 (26.2%)	272/601 (45.3%)
B: rec-FSH			
	Statistics	T2 vs. T3 > 50% decrease LH	LH < 1.2 IU/l at T3
No	n/N (%)	209/286 (73.1%)	239/430 (55.4%)
Yes	n/N (%)	77/286 (26.9%)	192/430 (44.6%)
C: hp-hMG			
	Statistics	T2 vs. T3 > 50% decrease LH	LH < 1.2 IU/l at T3
No	n/N (%)	87/115 (75.6%)	91/171 (53.2%)
Yes	n/N (%)	28/115 (24.4%)	80/171 (46.8%)

A decrease of LH >50% between T2 and T3 or LH level < 1.2 IU/l indicates 'oversuppression'. A. All gonadotrophins. B. Rec-FSH. C. hp-hMG.

**Table 6:** Odds ratio for the impact of confounding factors based on patient characteristics (Anti-Müllarian hormone, age and Body Mass Index) after logistic regression analysis on LH <1.2 IU/L at T3 or >50% decrease of LH between T2 and T3.

Patient characteristics	T2 vs. T3 > 50% decrease LH		LH < 1.2 IU/l at T3	
	Odds Ratio (95% CI)	*p-value*	Odds Ratio (95% CI)	*p-value*
Anti-Müllerian Hormone	1.03 (0.96;1.10)	0.47	1.00 (0.94;1.06)	0.93
Age	0.95 (0.90;0.99)	**0.02**	0.91 (0.88;0.94)	**<0.01**
Body Mass Index (BMI)	0.94 (0.88;0.99)	**0.03**	1.00 (0.96;1.05)	0.86

Bold: indicates significant impact of patient characteristic.

**Table 7:** Positive pregnancy test after fresh embryo transfer in antagonist cycles regarding the effect of oversuppression. A decrease of LH > 50% between T2 and T3 or LH levels <1.2 IU/l indicate 'oversuppression'.

A: all gonadotrophins					
		T2 vs. T3 > 50% decrease LH		LH < 1.2 IU/l at T3	
	Statistics	Yes	No	Yes	No
hCG +	n/N (%)	11/43 (25.6%)	67/173 (38.7%)	48/132 (36.4%)	62/185 (33.5%)
B: rec-FSH					
		T2 vs. T3 > 50% decrease LH		LH < 1.2 IU/l at T3	
	Statistics	Yes	No	Yes	no
hCG +	n/N (%)	7/31 (22.6%)	51/115 (44.4%)	31/85 (36.5%)	46/123 (37.4%)
C: hp-hMG					
		T2 vs. T3 > 50% decrease LH		LH < 1.2 IU/l at T3	
	Statistics	Yes	No	Yes	no
hCG +	n/N (%)	4/12 (33.3%)	16/58 (27.6%)	17/47 (36.2%)	16/62 (25.8%)

A. All gonadotrophins. B. Rec-FSH. C. hp-hMG.

In 637 antagonist cycles a blood sample was taken at T2 and in 601 antagonist cycles a blood sample was taken at T3, i.e. 24 hours after GnRH antagonist initiation. When the cut-off level of 1.2 IU/L to define oversuppresion, was applied for T2, in 166 (26.06%) antagonist cycles LH was lower than 1.2 IU/L. However, the highest proportion of low LH levels was seen at T3 after GnRH antagonist initiation, with a drop of LH level below 1.2 IU/l in 45.3% cycles (272/601). In 192 antagonist cycles (192/430; 44.7%) with rec-FSH, LH was lower than 1.2 IU/l after GnRH antagonist initiation, compared to 80 cycles (80/171; 46.8%) with hp-hMG (**Table 5**). Logistic regression analysis to evaluate possible confounding factors for LH oversuppression with an LH <1.2 IU/L at T3 only showed an significant odds ratio for age (OR 0.91 (0.88;0.94), P <0.01). However, BMI (OR 1.00 (0.96;1.05), P =0.86), and AMH did not impact (OR 1.00 (0.94;1.06), P = 0.93) LH levels < 1.2/L (**Table 6**). In 317 cycles a fresh embryo transfer was performed resulting in a positive pregnancy test in 36.4% (48/132) of cycles in the oversuppressed group compared to 33.5% (62/185) in the group where LH remained > 1.2 IU/L at T3. Pregnancy rates using rec-FSH lead to a rate of 36.5% (31/85) in the oversuppressed group, compared to 37.4% (46/123) in the group where LH remained > 1.2 IU/L at T3. Using hp-hMG pregnancy rates in the oversuppressed group were 36.2% (17/47) and 25.8% (16/62) in the group where LH remained > 1.2 IU/L at T3 (**Table 7**).

## 4 Discussion

To the best of our knowledge, our study is the first to describe the course of LH levels during ovarian stimulation in a short-agonist and antagonist protocol. Our results confirm an initial increase of LH after start of GnRH-analogue administration at start of ovarian stimulation in a short-agonist protocol, hence also referred to as a ‘micro-flare’ protocol. Subsequently, LH levels decrease gradually and remain low until the day of oocyte maturation triggering. On the other hand, during stimulation according to the more commonly used antagonist protocol, a decrease in LH levels was observed as of initiation of gonadotrophins, with a further steep drop in LH levels after administration of the GnRH antagonist. Thereafter, LH levels increased significantly until oocyte maturation triggering, although absolute levels remained low.

Several studies in long-agonist protocols have described a detrimental effect of severe LH suppression on live birth rates (7). In a dose-finding study on GnRH antagonist, a critical role of LH on implantation has been suggested because of a trend of lower clinical pregnancy rates in patients with the lowest LH levels, since the number of follicles, oocytes, quality of oocytes, number and quality of embryos were not significantly different between the studied groups (20). Others have hypothesized that a certain level of LH is required to maintain androgen and subsequently, estradiol production for follicular development during COS (21, 22). Likewise, the clinical phenomenon of ‘oversuppression’ of LH was introduced, referring to a relative LH deficiency within 24 hours after GnRH antagonist administration and subsequent inadequate increase of estradiol levels (17). Kol et al. described oversuppression, 24 hours after GnRH antagonist administration, as LH levels that dropped >50% of pre-injection levels (17). According to the “LH window” concept outlined by Shoham, a 1.2 IU/l threshold level of serum LH should be reached to ensure sufficient estrogen production leading to normal follicular development, endometrial proliferation and corpus luteum formation.

Our data allowed us to compare LH levels during stimulation with different types of gonadotrophins. Despite the structural differences between rec-FSH and hp-hMG, the pattern of change in LH during ovarian stimulation was similar. One can hypothesize that addition of hp-hMG may be an option to prevent low LH levels during stimulation. Multiple studies have shown that the use of hp-hMG, containing hCG and thus LH-activity, was not inferior to recFSH in terms of live birth after ART (3). In our study, changes in LH levels during ovarian stimulation cycles with recFSH and in cycles with hp-hMG followed a rather similar pattern. In addition, the percentage of cycles with oversuppressed levels did not seem to differ between rec-FSH and hp-hMG. These findings suggest that recFSH and hp-hMG seem to have a similar impact on the course of LH levels during ovarian stimulation, and thus on outcomes of stimulation.

In our data we applied aforementioned criteria for LH oversuppression. In up to 26.2% of cycles, a drop in LH > 50% after initiation of GnRH antagonist was observed and in no less than 45.3% of all cycles LH levels dropped below 1.2 IU/l within 24 hours after GnRH antagonist initiation. Hence, our findings indicate that LH oversuppression may be more ubiquitous than suggested by previous studies (17, 19). Until so far, the clinical relevance of LH oversuppression needs to be confirmed. The similar percentage of cycles with low LH levels after GnRH antagonist during COS with hp-hMG as compared with rec-FSH, suggest that LH oversuppression may not have a clinical impact. On the other hand, our data may also suggest that LH-activity in hp-hMG is insufficient to prevent a sharp decrease of LH after GnRH antagonist administration. Unfortunately, data from prospective randomized studies comparing hp-hMG to recombinant FSH + recombinant LH on outcomes of ovarian stimulation and pregnancy are not available. Nevertheless, administration of recombinant LH has been proposed to avoid the so-called oversuppression of LH and to improve outcome (5, 23). More specifically, in women 36-39 years of age, rec-LH supplementation may exert a beneficial effect on implantation rates regardless of pituitary suppression protocol (2). In our study, younger patients were more at risk of LH levels < 1.2 IU/L and a decrease of LH by >50% after GnRH antagonist initiation. Lower BMI levels also had a significant impact on the risk of >50% decrease of LH. This seems contradictory with the aforementioned studies suggesting that rec-LH may be needed in treatment of women age 36-39 years old. Also women with a unexpected low response to FSH monotherapy and GnRH agonist-induced pituitary down-regulation appeared to benefit from rec-LH supplementation (2). However, in none of these studies LH levels were taken into account, i.e. absolute LH levels were not assessed and thus, it remains unclear at what LH level supplementation may improve ART outcomes. Our data are similar to the study by Depalo et al., who observed lowest LH levels after COS in antagonist protocol in young women with good ovarian response who achieved a pregnancy as compared to women who did not get pregnant (14). In our study, the number of positive pregnancy tests was slightly higher in cycles with an LH < 1.2 IU/L (36.4%) and subsequent fresh embryo transfer compared to the group that had an LH > 1.2 IU/L (33.5%). In addition, young women from our study were more prone to have low LH levels. However, women with a low BMI were also more prone for oversuppression, whereas AMH levels did not impact the risk of low LH levels. Moreover, in a considerable part of cycles included in our study, LH levels were profoundly suppressed during COS, even prior to GnRH antagonist initiation at T2 (26.1%). Therefore, our data need to be interpreted with care. This was a retrospective single center study. No analysis was performed to adjust for other possible confounding factors because of the descriptive intent of the study. For the sub-analysis on oversuppression of LH, data were retrieved from a selected number of ovarian stimulation cycles in antagonist protocol alone, during which hormonal measurements had been performed on the day of antagonist initiation and 24 hours later, cycles of a duration of 9 to 16 days and followed by a fresh embryo transfer. Because of the limited number of cycles and thus, even lower number of patients, life birth rates were not calculated and the clinical significance of our findings need to be confirmed in larger data sets to draw firm conclusions on the meaning of low LH levels during ovarian stimulation.

## 5 Conclusion

In this retrospective study we describe the course of LH during ovarian stimulation cycles in a short-agonist and in an antagonist protocol. Regardless of the type of gonadotrophin administered, LH seems to follow a similar pattern from the midfollicular phase until oocyte maturation trigger. Based on the current findings and earlier described criteria, LH oversuppression may be a rather pervasive phenomenon during ovarian stimulation with an antagonist protocol and warrants further investigation to elucidate the clinical relevance of low LH levels with regard to IVF treatment outcome.

## Data Availability

The raw data supporting the conclusions of this article will be made available by the authors, without undue reservation.
